# Role of Family Motivation, Workplace Civility and Self-Efficacy in Developing Affective Commitment and Organizational Citizenship Behavior

**DOI:** 10.3390/ejihpe10010027

**Published:** 2020-01-07

**Authors:** Humaira Erum, Ghulam Abid, Francoise Contreras, Talat Islam

**Affiliations:** 1 School of Business Administration, National College of Business Administration & Economics, Lahore 54000, Pakistan; drghulamabidl@gmail.com; 2 Escuela de Administración, Universidad Del Rosario, Bogotá 111711, Colombia; francoise.contreras@urosario.edu.com; 3 Institute of Business Administration, Uiversity of the Punjab, Lahore 54000, Pakistan; talatislam@yahoo.com

**Keywords:** family motivation, civility, self-efficacy, affective commitment, organizational citizenship behavior

## Abstract

The mechanism connecting the antecedents to positive attitudes like affective commitment (AC) and positive behaviors like organizational citizenship behavior (OCB) is an under-researched area in the field of positive organizational scholarship. Drawing on Social Exchange Theory (SET), this study empirically validates family motivation and civility as antecedents of affective commitment and organizational citizenship behavior through the mediating mechanism of self-efficacy. The process by Hayes (2013) was used to analyze time-lagged and multi-source data collected from 335 employees of educational and telecom sector. Results indicate that the relationship of affective commitment with family motivation and civility is partially mediated whereas the relationship of organizational citizenship behavior with family motivation and civility is fully mediated by self-efficacy. This study adds to the literature of family-work enrichment accounts by validating family motivation as a novel antecedent for positive behavioral outcomes. The implications of the study are discussed.

## 1. Introduction

In the dynamic work environment, it is really challenging for the management to find and retain dedicated and committed employees who identify themselves with the organization [1,2]. Organizations need committed employees who go an extra mile to perform tasks that are not formally defined in the job description but are beneficial for the effective functioning of the organization. Empirical studies have shown that organizations having a workforce with positive attitudes and behaviors outshine as compared to those who don’t have such employees [3]. Existing literature indicates that theoretically and practically it is important to investigate that to what an extent personal and contextual factors determine employee’s values, behaviors [4,5] and his/ her psychological attachment to work [6,7]. That is why, a lot of attention has been paid to empirically validating the antecedents of positive outcomes like organizational commitment and organizational citizenship behaviors (OCBs) [8,9,10,11,12]. Although existing literature has substantiated the antecedents of affective commitment and organizational citizenship behavior, still more attention is required to establish the mechanism through which these antecedents are connected to affective commitment [13] and organizational citizenship behavior [14].

Today’s workplace is characterized by many stress factors like high job demands, insufficient job resources, work overload, ambiguity [15], high competitiveness, change [16,17] workplace bullying, lack of social support, working conditions [18]. Moreover, in the developing countries, where job opportunities are scarce, people are bound to do whatever jobs they get, jobs are usually underpaid and there are many repetitive and routine jobs that provide little skill variety and autonomy. All these factors contribute to lower intrinsic and extrinsic motivation of the employee. In this scenario, the question arises that what keeps the employees working and why they show positive attitudes and behaviours for an organization? We argue that family motivation and civility play its role. Employees usually work to support themselves and their family [19,20]. Therefore, it may be a motivational force for them to be a good soldier and stay committed with the organization. Existing literature mainly focuses on work-family conflict [21,22,23] and identify family as a meddling factor in employee’s work life. There are relatively fewer studies that recognize the role of family to support and energize work life [24,25] and enhance job performance [19,26]. Family motivation is an emerging concept that requires theoretical and empirical research for substantiating its determinants and outcomes. It is reported, “….in terms of the consequences of family motivation, we encourage future researchers to examine factors beyond job performance, such as organizational commitment and organizational citizenship behaviors” [19].

On the other hand, civility at workplace is identified as driver of positive individual and organizational outcomes [27]. Civility at workplace refers to employees’ courteous, respectful and caring behavior towards each other in formal and informal social relations [27]. It is not only being polite but being able to disagree without being disrespectful. It is the tool to solve problems and discuss ideas [28]. Civility fosters positive outcomes like thriving, performance, organizational citizenship behavior and health [27,29] and has its importance in customer service, problem solving, team building, relationship and trust building [28]. Despite the physiological, psychological and relational benefits of civility at the workplace, few empirical studies have concentrated the beneficial outcomes of civility in management literature [27]. Moreover, civilty is culture and time sensitive as what is considered civilized at one time in a particular culture may not be so in the other culture at some other time period [28]. Therefore, construct of civility is required to be addressed according to time, situation, culture with due importance given to acceptable standards. In response to the above gaps in the literature, the present research identifies civility at the workplace as the determinant of affective commitment and organizational citizenship behavior. More importantly, this study explores the mechanism through which family motivation and civility result in aforementioned positive consequences by examining the mediating role of self-efficacy.

We argue that employee views the provision of job by the organization as a means to fulfill their need of supporting the family and enhancing their confidence towards their abilities and skills so they feel obligated to reciprocate by being committed to the organization and performing extra-role behaviors. Similarly, experiencing respect and civility at the workplace also indebt employees to show positive behavior towards organization and coworkers (OCBs). These arguments have been developed on the basis of Social Exchange Theory (SET) [30] which states that, when one party receive something valuable, it tries to reciprocate to the giver with similar or greater values. Such relations are developed on the basis of norms of reciprocity [31].

It is also important to mention that the majority of the studies on civility and family motivation has been conducted in Western countries [19]. Therefore, researchers have emphasized investigating the family motivation and civility in non-western country’s setting (i.e., Pakistan) for generalizability of the theories across regions [32]. The family is considered as an integral part of the Pakistani culture as it has a conventional family system where each member has a certain commitment towards other members of the family [33]. Such characteristics made Pakistani culture as collectivist, hence family is considered as their priority [34]. Therefore, understanding family motivation and civility in Pakistani culture add to the existing literature on the work-family association. Given that, the first objective of this study is to examine the mechanism through which family motivation is related to affective commitment and organizational citizenship behavior, and the second objective is to examine the mechanism through which civility is related to affective commitment and organizational citizenship behavior, and the second objective is to examine the mechanism through which civiliaty is related to affective commitment and organizational citizenship behavior.

Our research makes the following contributions. First, it contributes to motivation and work-family dynamics by responding to the call for examining the outcomes of family motivation. Second, it provides empirical evidence from a non-western country to establish the consequences of family motivation and civility. Third, our study extends the existing knowledge on work-family research by identifying a novel path through which family can supplement positive attitude and behaviors to enhance organizational effectiveness.

This study is conducted on population from service industry and included eemployees from education and telecom sector. The rationale behind selecting teachers is that teaching is considered as an underpaid profession in developing countries and frontline employees lack skill variety and autonomy due to repetative and routine nature of tasks, so, intrinsic and extrinsic motivation is expected to be low in this population. Moreover, the teacher are not only required to perform their job descriptions but also are required to perform certain tasks beyond job description [35] like publishing research in well-reputed journals to uplift the name of their institute or event management or counseling/training students [15]. Similarly, frontline employees are essential in creating value, handling customers’ complaints and service delivery [36]. These factors contribute to increase workloads and stress levels further reducing intrinsic motivation.

## 2. Literature Review

Researchers have recognized the role of employees’ positive attitudes and behaviors for effectiveness, efficiency and sustainable competitive advantage to an organization [37,38]. Seminal work of Katz et al. [39] recognized that effective organizational functioning requires three factors, i.e., organizational commitment, in-role job performance and extra-role behaviors. Extra-role behavior also termed as OCB, which is defined as “discretionary individual behaviors that are not explicitly defined in the job description and are not formally rewarded but are essential for the effective and efficient functioning of an organization” [40]. OCB is one of the most influential constructs in last twenty years as depicted by comprehensive literature reviews [9,36,41]. Literature is evident that leadership style, in-role clarity, organizational justice, individual traits [42], job satisfaction, organizational commitment [43], perceived fairness and personality dimensions [40] are major antecedents of OCB. Although much is known about the antecedents of organizational citizenship behavior, little is known about why employees engage in OCB, their underlying motives [14]. We argue that family motivation and workplace civility through the mediating mechanism of self-efficacy acts as a motivator for employees to engage in such behaviors.

Similarly, affective commitment referring to employee’s sense of belonging and emotional bonding to his/her organization undermine many negative behaviors like absenteeism and turnover etc. [44] and foster positive attitudes like job involvement, work engagement, and enhance positive behaviors like organizational citizenship behavior and performance [45]. The current attention of researchers towards work-family enrichment, as opposed to work-family conflict, suggests that work-family enrichment results in positive outcomes like performance, satisfaction, well-being, affective commitment [46]. However, less is known about how this work-family enrichment generate these positive outcomes [47]. This study addresses the gap by suggesting the mediating role of self-efficacy on the relationship between family motivation and affective commitment.

### 2.1. Family Motivation, Self-Efficacy, Affective Commitment and Organizational Citizenship Behavior

Family is defined as “people related by biological ties, marriage, social customs or adoption” [48] and family motivation refers to “the desire to expend effort to benefit one’s family” [19]. Family motivation is a variant of but different from pro-social motivation in following ways. First, for family motivation, the beneficiary is one’s family, whereas, pro-social motivation focuses on co-workers, customers or some particular group [49]. Second, family motivation is not related to the job and is expected to be consistent in different contexts whereas pro-social motivation is task or job specific. Third, family motivation is expected to be more intense than pro-social motivation because of the one’s deep concern for his/her family [19]. Still, it is theoretically and empirically an under-investigated area [20]. The positive side of work-family relationship commonly known as ‘work-family enrichment accounts’ need researchers’ attention for specifying their underlying mechanism [19,50]. We argue that family motivation is a strong predictor of affective commitment and organizational citizenship behavior and is connected through the mediating role of self-efficacy as employees view their work as a way to improve their skills and as source to provide for family needs. Self-efficacy is the employee’s belief about his/her capability to perform effectively to attain positive outcomes [35]. Self-efficacy signifies judgments that how well the person can perform an action, which in turn might affect individual’s adoptions of action, how much effort he or she will spend, and how long he or she will continue in that situations [6,35]. Self-efficacy is considered as context-dependent and situation specific construct that acts as a “cognitive mediator of actions” [51]. Literature is well documented about the mediating role of self-efficacy between certain variables. For example, literature provides strong evidence for mediating role of self-efficacy between motivation and performance, between transformational leadership and creativity [52], affective well-being and extra-role performance [53]. Moreover, employee job satisfaction, motivation, learning intention, productivity, and career commitment [54] have been identified as positive outcomes of self-efficacy. Self-efficacious individuals are known for their high persistence and determination in difficult situations that brings them high success. In addition, self-efficacious individuals might be working hard toward innovative solutions in their organizations, therefore, their innovative ideas might be increasing their performance directly [6].

When employees perceive their work as a meaningful activity to serve the family, recognize that they can fulfill family needs through their work, and their family also appreciates their effort for benefitting it, then they experience the feelings of self-worth and develop confidence in their skills and abilities, that is, they become more self-efficacious. According to the SET [30], outcomes are based on a combination of parties’ efforts and mutual and complementary arrangements. Individuals weigh the cost and potential benefits of social relationships, seeking cost minimization and benefit maximization. Researchers have identified perceived organization support, leader-member exchange and trust as a relational mechanism to facilitate social exchange. We consider family motivation and civility as an exchange mechanism based on reciprocity. When employees believe that their work is a source to serve family needs, they not only feel obligated but also feel emotionally attached to pay back to the organization in terms of positive attitudes and behaviors [19]. However, in order to perform better, positive perception about ones’ abilities is very essential [33]. Literature has a strong indication that people who are self-efficacious outperform those who feel doubtful about their abilities and skills to perform a particular task [55]. They exert more effort toward their work [56]. Conceiving work as a way to benefit the family, they remain emotionally committed to the work (affective commitment). Self-efficacy as an important direct and indirect determinant of the development and maintenance of the employee-organization relationship [28]. It directly influences employee’s cooperation and effort. Self-efficacy also involves a motivational potential towards work behaviors which were mainly contributive to their organizations [6]. Self-efficacious employees perform not only in-role activities but also engage themselves in extra-role behaviors (OCB) that benefit the organization [57]. So we propose that;

**Hypothesis** **1.** *Self-efficacy mediates the relationship between family motivation and affective commitment.*

**Hypothesis** **2.** *Self-efficacy mediates the relationship between family motivation and organizational citizenship behavior.*

### 2.2. Civility, Self-Efficacy, Affective Commitment and Organizational Citizenship Behavior

Civility refers to polite, respectful and considerate behavior toward others [28]. Employees’ expectation of respectful treatment from colleagues, customers and management makes civility the ultimate quality of an organizational culture [58]. For this study, we take into account the definition of [59] which states that civility is “claiming and caring for one’s identity, needs and beliefs without degrading someone else in the process” Acceptable standards of civility do vary from one generation and/or culture to the next and civility appears in various forms. These standards depend on changing norms and traditions of the a society/culture and are manifested not only in folks and families but also in organizational culture [28]. At workplace, where culture, norms, values, mores, and traditions act as boundaries for the way people treat one another, civility is a valuable competency. This means that civility is a competency, “a specific personal characteristic which contributes to effective and/or superior performance” [28] such as motivation, self-knowledge, willingness to perform and other measurable, observable attitudes and behaviors that can be developed in individuals and teams. In recent years, some dominant and respected business practitioners have recognized the importance of civility in their companies and they care that their companies employed civilized individuals. One of the reason is that if employees are not civilized to customers, businesses lose market share. If they cannot work well together, the organizational culture becomes toxic, biased, and the company develops blind spots where problems grow, rather than opportunities for growth [28].

Civility not only buffers against stress, burnout and counter-productive behaviors, but also engenders positive feelings, work attitudes, trust, helpful behaviors, and well-being [27]. In contrast, uncivil behavior at the workplace creates issues by generating negative outcomes, both at an individual and organization level like stress, depression, lost productivity and even retaliation against the organization [27,60].

Despite being a beneficial aspect of the workplace, civility has been an empirically under-focused area in the field of management [27]. We focus that, when employees experience respectful, considerate and polite interactions from management, coworkers and customers, they increasingly feel as valued organizational members. These experiences enhance their efficacy feelings and and they feel high about their skills and capabilities at work. When employees are treated respectfully, based on the reciprocity norms of SET, employees feel inclined to respond through positive attitudes and behaviors. They feel emotionally attached and being identified by organization values (affective commitment). So it is comprehendible to say that;

**Hypothesis** **3.** *Self-efficacy mediates the relationship between civility and affective commitment.*

Considering oneself as a capable and efficacious member of the organization, the employee usually cares for the co-workers, tries to help them in their work. Similarly, an employee’s identification and attachment to the organization push him/her to indulge in discretionary behaviors like expressing loyalty towards the organization and protecting the organization from potential harms. Conversely, the employees who face incivility at the workplace feel low on his/her capability to perform and does not offer any help to coworkers as well as the organization. Therefore, we hypothesize that;

**Hypothesis** **4.** *Self-efficacy mediates the relationship between Civility and organizational citizenship behavior.*

**Figure 1:**
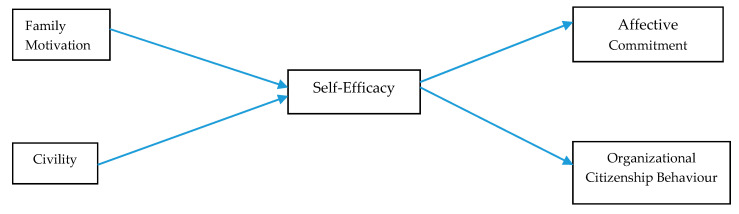
Theoretical Model.

The theoretical model is shown below in Figure 1.

## 3. Methodology

### 3.1. Participants and Procedure

For the empirical analysis, the study approached employees working in education and telecom sector. First, we recruited and trained a group of 10 research students regarding the data collection procedure and intricacies of research to minimize the researcher interference. These students visited the different institutions and telecom organizations for data collection. These students explained the purpose of research and asked their consent to participate in a research study. The anonymity and confidentiality of information was assured and communicated to every respondent. A letter from the institution was issued to ensure the confidentiality of responses. Given the reliance on the validated survey instrument for data collection, those who consented to participate were sampled in three sessions (time lagged) separated by 7 days approximately to reduce common method bias as suggested by [61]. Employees were scheduled for 20 min meeting from organization paid time. The employees provided a self-reported response about demographics, motivation to work and civility at time period 1 (T1). The data for self-efficacy and affective commitment was taken at time period 2 (T2). The respondents were requested to provide a colleague’s name who was contacted at time period 3 (T3) to rate respondent’s OCB. The data collection procedure was identical at each stage of the data collection period. At time period 1, a total of 450 questionnaires were distributed. Out of those, 382 completed questionnaires were received, making a response rate of 84.8%. The respondents were requested to write their names or self-identifiable code on the questionnaire for matching data at Time 2. At T2, questionnaires were distributed to all the 382 participants who responded at T1 to provide data regarding self-efficacy and affective commitment. Also, at T3, colleagues of 382 participants who responded at T1 were contacted to get data regarding OCB of the participants. Out of the 382 who responded at T1, only 355 participants responded at T2 and colleagues of 362 participants responded at T3. After cross matching the respondent’s and colleague’s information and eliminating the incomplete and wrong questionnaires, we had an actual sample of 335, hence making a response rate of 74.4%. We have used the responses of only those respondents in the analysis, whose data was complete in respect of all study variables. As regards to the adequacy of sample size, in behavioural research, sample size of between 30 and 500 is recommended (see for example, Roscoe, 1975, p. 163 or Abranovic, 1997, pp. 307–308). As a rule of thumb, Rosocoe (1975) indicates sample size should be at least ten times larger than the number of variables being considered in multivariate analysis.

A group of 335 employees who participated in the study, consisted of 195 males (58%) and 140 females (42%). The average age of the respondents was 31 years (SD = 8.11). Most of the participants were holding postgraduate degree (40%), followed by graduate degree (33%). Of the total of 335 respondents, 152 (45%) were single and 183 were married (55%) with the average tenure of 5 years with the organization (See Table 1).

### 3.2. Measurement

We used adapted questionnaire from the previous studies as they were already examined regarding their internal consistency. The internal consistency of all the scales used in this study were also noted above the standard value of 0.70 [62].

A five-item scale of the Ryan et. al. [63] was used to examine family motivation on a five-point Likert scale ranging between “1 = strongly disagree to 5 = strongly agree.” Sample items include “My family benefits from my job?” and “It is important for me to do good for my family?” The internal consistency of the scale of our data set was 0.84. Civility refers the extent to which employees behave respectfully, politely showing dignity to each other, which was measured through [64] four-item scale on a five-point Likert scale (i.e., 1 = never to 5 = always). Sample items include “Do your co-worker treat you with respect?” and “Do your co-worker treat you with dignity?” This study noted 0.77 as the value of its internal consistency.

The self-efficacy of the employee was assessed using an eight-item scale of [65]. Respondents were evaluated on a five-point Likert scale ranging between “1-never to 5-very often”. The internal consistency of this scale was noted as 0.85. A sample item includes, “When facing difficult tasks, I am certain that I will accomplish them.” Similarly, affective commitment was measured using a three-item scale of [66] on five-point Likert scale (i.e., “1 = strongly disagree to 5 = strongly agree”). The internal consistency of this scale was noted as 0.76. A sample item is, “I feel loyal to this organization.”

Finally, OCB was measured using [67] eight-item on a five-point Likert scale ranging between “1-never to 5-every time”. The study noted 0.84 as the value of its internal consistency with a sample item of, “show pride when representing the organization in public.”

Literature suggested that age, qualification and tenure of the employees have a significant association with OCB and affective commitment [35,68,69] and civility [70] therefore, these are considered as control variables.

Analytical Strategy: The hypothesized model was tested in two steps. In the first step, parcels of the items were made to check the measurement model [71]. Second, the hypothesized model was tested using a process by Hayes [72]. The full measurement model was tested and compared with alternate models using traditional good indices: goodness-of-fit index (GFI) and the comparative fit index (CFI), Tucker-Lewis index (TLI) and root mean squared error of approximation (RMSEA) [73]. The acceptable value for RMSEA is <0.08 and for other indices >0.90.

Total ten parcels of all items related to three study constructs were created. A parcel refers to an ‘aggregate-level factor, including computing two or more items’ [74]. The measurement model created using parcels produce more reliable results [75] and also helps in removing Type I error [74]. In this study, two parcels of the four civility items, four parcels of eight OCBs items and four parcels of eight self-efficacy items were formed. Then, these three constructs along with affective commitment and family motivation were entered in the measurement model as latent factors.

In the second step, model 4 of process by Hayes with 5000 bootstrapping was used to assess the mediating effect of self-efficacy on the relationship between family motivation, civility and OCB and affective commitment. This technique calculates precise and correct confidence intervals of indirect effects as compared to the causal step strategy of [76].

To avoid common method bias [61] due to self-reported measure of four construct, Harman’s single factor test [77] was also performed using SPSS and conducting exploratory factor analysis on all self-reported items of study variables. The results indicate that four factors emerged instead of a single factor and the first factor explained only 20.01% variance which is below the 50% level. Also aggregate variance explained by all retained factors was 57.98%, which suggests that common method bias is not an issue in this study.

## 4. Results

To assess the construct validity, convergent and discriminant validity were estimated. According to [69], construct factor loadings, composite reliability (CR) and the average variance extracted (AVE) are required to be calculated. The acceptance criteria is that factor loadings for each variable should be greater than 0.60, composite reliability should be greater than 0.70 and average variance extracted should be greater than 0.50. For discriminant validity, ‘square root of AVE of each construct should be greater than the correlations of this construct to all the other constructs’ [78]. The results, presented in Table 2, show that the factor loadings are greater than 0.64. Moreover, all variables have CR and AVE greater than 0.70 and 0.50 respectively. Thus, fulfilling the criteria for convergent validity.

Confirmatory Factor Analyses (CFA) revealed that the hypothesized five factor model (χ2 = 236.36 with df = 125; RMSEA = 0.052; CFI = 0.95; TLI = 0.94, GFI = 0.92) substantially fits the data better than the other models like, for example, one-factor model A (χ2 = 2028.87 with df = 350; RMSEA = 0.12; CFI = 0.50; TLI = 0.46, GFI = 0.63) and three factor model C(χ2 = 1304.89 with df = 347; RMSEA = 0.09; CFI = 0.71; TLI = 0.69, GFI = 0.71) (see Table 3).

### 4.1. Preliminary Analysis

Initial support for proposed hypotheses is solicited using bivariate correlation among the variables. Table 4 presents the descriptive statistics and correlation among variables which provides support for further hypothesis testing.

Positive and significant correlation exists among the study variables. Family motivation is positively and significantly related to affective commitment (r = 0.28, *p* < 0.01), self-efficacy (r = 0.27, *p* < 0.01), and OCB (r = 0.17, *p* < 0.01). Correlation analysis also shows a positive and significant relationship between civility and affective commitment (r = 0.32, *p* < 0.01), civility and self-efficacy (r = 0.28, *p* < 0.01), and civility and organizational citizenship behavior (r = 0.14, *p* < 0.01) which provides support for direct relationships in the model. Moreover, a significant correlation between self-efficacy and affective commitment (r = 0.53, *p* < 0.01), and self-efficacy and organizational citizenship behavior (r = 0.41, *p* < 0.01) was found which supported direct relationships between mediator and criterion variable. Age, education level, and employee tenure in an organization are considered as controls. As results show age had significant positive correlation with family motivation (r = 0.16, *p* < 0.01); civility (r = 0.14, *p* < 0.01); commitment (r = 0.12, *p* < 0.05) [58] and organizational citizenship behavior (r = 0.11, *p* < 0.05) [59] which is in line with literature. Similarly, civility and education level are positively and significantly related (r = 0.12, *p* < 0.05) [61]. Literature shows support for positive significant correlation between tenure and affective commitment (r = 0.10, *p* < 0.05) [13], OCB (r = 0.12, *p* < 0.05) [69].

Positive and significant correlation exists among the study variables. Family motivation is positively and significantly related to affective commitment (r = 0.28, *p* < 0.01), self-efficacy (r = 0.27, *p* < 0.01), and OCB (r = 0.17, *p* < 0.01). Correlation analysis also shows a positive and significant relationship between civility and affective commitment (r = 0.32, *p* < 0.01), civility and self-efficacy (r = 0.28, *p* < 0.01), and civility and organizational citizenship behavior (r = 0.14, *p* < 0.01) which provides support for direct relationships in the model. Moreover, a significant correlation between self-efficacy and affective commitment (r = 0.53, *p* < 0.01), and self-efficacy and organizational citizenship behavior (r = 0.41, *p* < 0.01) was found which supported direct relationships between mediator and criterion variable. Age, education level, and employee tenure in an organization are considered as controls. As results show age had significant positive correlation with family motivation (r = 0.16, *p* < 0.01); civility (r = 0.14, *p* < 0.01); commitment (r = 0.12, *p* < 0.05) [35] and organizational citizenship behavior (r = 0.11, *p* < 0.05) [68s] which is in line with literature. Similarly, civility and education level are positively and significantly related (r = 0.12, *p* < 0.05) [70]. Literature shows support for positive significant correlation between tenure and affective commitment (r = 0.10, *p* < 0.05) [13], OCB (r = 0.12, *p* < 0.05) [69].

### 4.2. Mediation Analysis

#### 4.2.1. Family Motivation, Self-Efficacy, Affective Commitment and Organizational Citizenship Behavior

**Figure 2:**
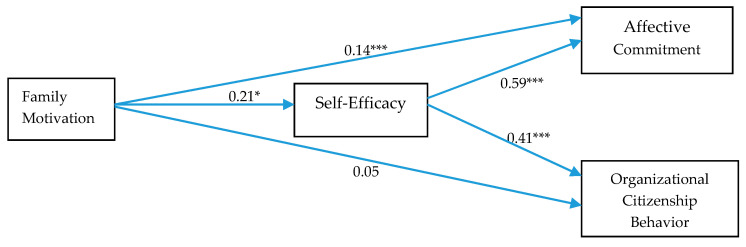
Path Coefficients. Notes: *** *p* < 0.001; * *p* < 0.05.

The Hypothesis 1 specifies that self-efficacy mediates the relationship between family motivation and affective commitment. To assess the mediating effect of self-efficacy on the relationship between family motivation and affective commitment, process by Hayes (2013), Model 4 was used with 5000 bootstrapping. The results are shown in Table 5 and Figure 2. Results indicate that family motivation significantly predicts both self-efficacy (β = 0.21, *p* < 0.001) and affective commitment with β = 0.14, *p* < 0.01, CI_95% confidence level_ (0.05, 0.23); self-efficacy also significantly predicts affective commitment (β = 0.59, *p* = 0.000). The significant indirect effect with β = 0.13 falling between 0.06 and 0.19 validates that self-efficacy mediates the relationship between family motivation and affective commitment. Hence, Hypothesis 1 is supported.

Hypothesis 2 was related to the mediated mechanism between family motivation and OCB. Results show that family motivation significantly predicts self-efficacy (β = 0.21, *p* < 0.001) but does not directly OCB, β = 0.05, *p* > 0.01, CI_95% confidence level_ (−0.02, 0.13); self-efficacy also significantly predicts OCB (β = 0.41, *p* < 0.001). For the indirect effect, β = 0.08 falls between 0.04 and 0.13. This range does not include zero; therefore, the indirect effect is significant, so the relationship between family motivation and OCB is mediated y self-efficacy. Hypothesis 2 is supported.

#### 4.2.2. Civility, Self-Efficacy, Affective Commitment and Organizational Citizenship Behavior

**Figure 3:**
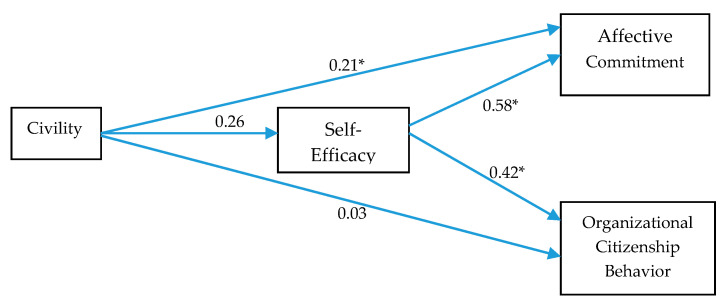
Path Coefficients. Notes: * *p* < 0.05.

It was predicted in Hypothesis 3 that self-efficacy mediates the relationship between civility and affective commitment. As shown in Table 6 and Figure 3, civility significantly predicts both self-efficacy (β = 0.26, *p* < 0.001) and affective commitment with β = 0.21, *p* < 0.01, CI_95% confidence level_ (0.10, 0.31); self-efficacy also significantly predicts affective commitment (β = 0.59, *p* = 0.000); self-efficacy also significantly predicts affective commitment (β = 0.58, *p* = 0.000). The positive β value for civility indicates that as civility increases, affective commitment also increases. Similarly, as self-efficacy goes up, affective commitment is also enhanced. The significant indirect effect with β = 0.15 falling between 0.09 and 0.22 validates that self-efficacy mediates the relationship between civility and affective commitment. Hence, Hypothesis 3 is supported.

Hypothesis 4 indicated that self-efficacy mediates the relationship between civility and OCB. Results in Table 6, show that civility significantly predicts self-efficacy (β = 0.26, *p* < 0.001) but does not directly impacts OCB, (β = 0.03, *p* > 0.001); self-efficacy also significantly predicts OCB (β = 0.42, *p* < 0.001).

For the indirect effect the β = 0.11 falls between 0.06 and 0.16. This range does not include zero; therefore, the indirect effect is valid and it is inferred that self-efficacy mediates the relationship between civility and OCB. The indirect effect is significant so the relationship between civility and OCB is mediated by self-efficacy and therefore, Hypothesis 4 is supported.

In addition, it is also evident from results that affective commitment has significant direct positive relationship with family motivation (β = 0.14, *p* < 0.05) (see Table 5) and civility (β = 0.21, *p* < 0.05) (Table 6) even when self-efficacy is entered in the model as mediator. Therefore, self-efficacy partially mediates the relationship between family motivation and affective commitment, and civility and affective commitment. It is shown in Table 5 and Table 6 that OCB does not have significant direct relationship with family motivation (*p* > 0.05) therefore, it is inferred that self-efficacy fully mediates the relationship between family motivation and OCB, and civility and OCB.

## 5. Discussion and Implications

Positive attitudes (affective commitment) and behaviors (OCB) are the most desirable qualities of the employees as these qualities translate into competitive advantage for the organization. Although the existing literature sufficiently substantiate for the antecedents of affective commitment and OCB, however, more research is needed to empirically validate the mechanism through which antecedents impact the positive behaviors. Attending this gap, we focused on empirically testing self-efficacy as the mediating mechanism between two antecedents i.e., family motivation, civility and two outcomes i.e., affective commitment and OCB. This study draws upon SET [30], and contributes theoretically to family-work enrichment literature by specifying family motivation as a novel antecedent of affective commitment and OCB. In line with the existing literature that validates positive outcomes of family motivation like energy and job performance [19], the results indicated that employees who perceive their work as a source to benefit their family, feel self-efficacious as they consider themselves capable of fulfilling their responsibility towards family. Since their work becomes the reason for enhancing their confidence about their skills/capabilities and satisfying family responsibility, in exchange, they get emotionally attached to the organization and perform extra role activities to benefit the organization. It is also indicated through results of the study that self efficacy partially mediates the relationship between family motivation and affective commitment; and civility and affective commitment. This suggests that if employee experience civilized behavior at workplace and view his work as means to benefit his/her family, he/she would develop emotional attachment with the job/organization. Moreover, his motivation and respectful treatment in organization makes him/her an efficious employee.

In addition to family motivation, civility is also empirically supported as a determinant of self-efficacy, affective commitment and OCB. Existing literature substantiates that civility at workplace fosters positive feelings [27] and buffers against the negative outcomes like stress, work deviance. Being treated respectfully, employees feel appreciated and highly valued, this positive feeling increases their self-concept, self-confidence and positively impacts their self-efficacy. Feeling self-efficacious and valued because of the considerate treatment by co-workers and management, in reciprocation, employees are obliged to remain committed to the organization [17] and indulge in activities that help the organization to work efficiently [53]. It is also noted in results that relationship of OCB with family motitation and civility is fully mediated by self-efficacy, which signifies that only those employees who feel high about his/her capabilities to handle tasks, achieve goals and solve work related problems, engage themselves voluntarily in extra-role tasks beneficial for organization. Those who do not feel self efficious, stick to in-role duties despite being viewing work as benefitting factor to support family and/or getting polite and considerate treatment at workplace.

The current study gains strength by adopting methodological measures to reduce common method bias as suggested by Podsakoff [61]. First, the anonymity was assured by assigning a self-identifiable code to the respondents. Secondly, different anchoring was used for scales of different variables and thirdly, the data for different variables was collected at three time periods separated by 14 days. Further, to avoid biases, observer (colleagues) assessed the OCB of the employees at time period 3, which provides strength to our research design.

Theoretically, this study adds to the literature by answering the call to empirically test the outcomes of family motivation [19] and explain the mechanism between affective commitment, OCB and their antecedents [14]. This study tests family motivation as the driver of affective commitment and OCB and also explains that self-efficacy partially mediates the relationship between family motivation, affective commitment and OCB. Moreover, self-efficacy fully mediates the relationship between civility, affective commitment and OCB. Thus, this study extends Blau’s social exchange theory by incorporating family motivation and civility as antecedents of commitment and extra-role behaviors.

From the individual perspective, the results of this study showed that family does not always conflict with work, but it can be the source of motivation to work. Moreover, family motivation and civility enhance one’s perception of his/her capabilities. Being efficacious, employees remain committed and act like a good citizen of the organization.

From the organizational perspective, managers always look for those employees who are committed and go an extra mile to make organization outperform competitors. Considering the positive outcomes as discussed, managers need to shape employees’ work orientation so that they consider work as benefitting their family. Practically, this study is helpful for human resource managers to devise family benefits, policies and promote a civilized and considerate culture that is imbued with family values to incline employees to show affective commitment and OCB. Moreover, our research is conducted in the collectivistic culture where employees are more inclined to take responsibility for their dependents, family motivation becomes more pertinent. Organizations may offer employees opportunities to meet their family needs. For example, offering flextime, providing a day-care facility at the workplace, providing assistance in children education/marriage or arranging family events may help the organizations win employees’ affective commitment to creating a win-win situation for both. Similarly, workplace civility can buffer against depression, stress and can be helpful in promoting the efficacy and well-being of employees that can translate into organizational and society’s well-being at large.

## 6. Limitations and Future Directions

Although the findings supported all the study hypotheses, the study still has certain limitation. First, our results are based on sample consisting of 335 employees from two service sectors and it may limit the generalizability of results. Future studies should work with larger diverse samples selected through probability sampling design to enhance the generalizability. Second, the use of cross-sectional data may not completely capture the true nature of the constructs like affective commitment, self-efficacy and family motivation as they represent one’s perception, emotion and psychological state respectively. Longitudinal data is more suitable for checking the psychological nature of constructs. As regards to the future research, more longitudinal studies and experiments are required to check causal and reciprocal relationships between the study’s constructs to enhance a deeper understanding of family motivation by explaining why and how it influences our work experience. Third, the current study uses survey design as it focuses on empirical evidence for organizational outcomes. Future research can take up family motivation phenomenon using case study method to consider socio-cultural context and whole value system of employee which will enhance theoretical understanding of the construct. Moreover, the possible darker side of family motivation like decreased voice behavior needs to be examined. In addition, there is a need to study the predictors and consequences of family motivation at organizational levels. Similarly, for affective commitment and OCB, future studies should focus on other mediators like grit, thriving and supervisory support. Moreover, moderators like intrinsic motivation, extrinsic motivation, gender, income level may be taken into account in future studies. From theoretical perspective, future research should focus on exploring different dimensions of family motivation like, for example, family motivation to support/benefit one’s family, family motivation to entail a sense of pride in one’s family. Moreover, in the existing literature, family motivation is taken up at a contextual level (reasons for action because of a particular life domain or a particular role, for example, a mother supporting her family by doing job) and further research is needed to explore it as a situational level (reasons for action that employee experience at specific moment in time). Like under special circumstances e.g death of a parent, some financial crises in family, some mishap with any sibling or child, break up with spouse.

## 7. Conclusions

Based on results of the study, it is concluded that family motivation emerged as a novel predictor of self-efficacy, affective commitment and organizational citizenship behavior but it is at nascent stage and needs further theoretical and empirical investigation. Similarly, civility at workplace can play an important role in producing positive individual and organizational outcomes. Family motivation, civility and self-efficacy as the determinants of positive attiitudes and behaviors help organization achieve competitive advantange.

## Figures and Tables

**Table 1:** Sample Profile.

Gender	Frequency	Percent
Female	195	58.2%
Male	140	41.8%
**Marital Status**		
Single	152	45.4%
Married	183	54.6%
**Qualification**		
Graduate	111	33.1%
Masters	134	40.0%
MPhil/MS	78	23.3%
Other	5	1.4%
**Age**		
Below 30	203	60.6%
31–35	63	18.8%
36–40	38	11.3%
41–45	10	3%
46–50	10	3%
Above 50	11	3%
**Organization**		
University	86	25.6%
College	56	16.7%
School	108	32.2%
Other (Telecom)	85	25.3%
**Tenure in Current Organization**		
Less than 5 years	81	24.1%
6–10	71	21.1%
11–15	65	19.4%
16–20	76	22.7%
More than 20 years	42	12.5%

**Table 2:** Construct Validity.

	Factor Loadings	CR	AVE	1	2	3	4	5
AC	0.64–0.81	0.76	0.51	0.72 *				
FM	0.70–0.82	0.84	0.52	0.34	0.72 *			
OCB	0.74–0.83	0.82	0.54	0.49	0.20	0.73 *		
Civ	0.85–0.87	0.72	0.57	0.43	0.23	0.20	0.75 *	
SE	0.73–0.80	0.83	0.55	0.66	0.33	0.49	0.36	0.74 *

Notes: * Values in diagonal represent the squared root estimate of AVE. AC = Affective Commitment, FM = Family Motivation, OCB = Organizational Citizenship behavior, Civ = Civility, SE = Self-Efficacy.

**Table 3:** Confirmatory Factor Analysis.

Models	χ^2^	df	X^2^/df	GFI	AGFI	CFI	TLI	RMSEA
Full Measurement Model	236.36	125	1.89	0.92	0.89	0.95	0.94	0.05
Model A ^a^	2028.87	350	5.79	0.63	0.57	0.50	0.46	0.12
Model B ^b^	876.82	344	2.54	0.82	0.79	0.84	0.82	0.06
Model C ^c^	1304.89	347	3.76	0.75	0.71	0.71	0.69	0.09
Model D ^d^	1725.66	349	4.94	0.68	0.62	0.59	0.56	0.11

Notes: ^a^ All constructs combined into one factor. ^b^ 4-factor model, FM, OCB, SE and AC and Civ combined as one factor. ^c^ 3-factor model, SE, AC + FM combined and OCB + Civ combined as one factor. ^d^ 2-factor model, FM + SE combined and OCB + AC + Civ combined as one factor.

**Table 4:** Descriptive and Correlations.

Variable	Mean	SD	1	2	3	4	5	6	7
Age	30.99	8.03							
Qualification			0.11 *						
Tenure	5.48	6.01	0.43 **	0.07					
FM	4.10	0.76	0.16 **	0.01	0.09				
Civ	4.39	0.64	0.14 **	0.12 *	0.07	0.19 **			
AC	4.05	0.72	0.12 *	0.02	0.10 *	0.28 **	0.32 **		
SE	4.02	0.59	0.09	0.06	0.06	0.27 **	0.28 **	0.53 **	
OCB	3.91	0.61	0.11 *	0.03	0.12 *	0.17 **	0.14 **	0.40 **	0.41 **

Notes: ** *p* < 0.01; * *p* < 0.05. AC = Affective Commitment, FM = Family Motivation, OCB = Organizational Citizenship behavior, Civ = Civility, SE = Self-Efficacy.

**Table 5:** Path Coefficients and Indirect Effects.

	Path Coefficients			Indirect Effect		
	SE	AC	OCB	Estimates	Boot LLCI	Boot ULCI
From → To						
Family Motivation (FM)	0.21 ***	0.14 ***	0.05			
Self-Efficacy (SE)		0.59 ***	0.41 ***			
**Indirect effect**						
FM → SE → AC				0.13	0.06	0.19
FM → SE → OCB				0.08	0.04	0.14
**Direct effect**						
FM → AC				0.14 ***	0.05	0.23
FM → OCB				0.05	−0.02	0.13
**Total effect**						
FM → AC				0.27 ***	0.17	0.36
FM → OCB				0.14 ***	0.05	0.22

Notes: AC = Affective Commitment, FM = Family Motivation, OCB = Organizational Citizenship behavior, Civ = Civility, SE = Self-Efficacy. *** *p* < 0.001.

**Table 6:** Path Coefficients and Indirect Effects.

	Path Coefficients			Indirect Effect		
	SE	AC	OCB	Estimates	Boot LLCI	Boot ULCI
From → To						
Civility (Civ)	0.26 ***	0.21 ***	0.03			
Self-Efficacy (SE)		0.58 ***	0.42 ***			
**Indirect effect**						
Civ → SE → AC				0.13	0.09	0.22
Civ → SE → OCB				0.15	0.06	0.16
**Direct effect**						
Civ → AC				0.21 ***	0.10	0.31
Civ → OCB				0.05	−0.06	0.13
**Total effect**						
Civ → AC				0.36 ***	0.24	0.47
Civ → OCB				0.14 ***	0.03	0.24

Notes: AC = Affective Commitment, FM = Family Motivation, OCB = Organizational Citizenship behavior, Civ = Civility, SE = Self-Efficacy. *** *p* < 0.001.
